# Generating Time-Series LAI Estimates of Maize Using Combined Methods Based on Multispectral UAV Observations and WOFOST Model

**DOI:** 10.3390/s20216006

**Published:** 2020-10-23

**Authors:** Zhiqiang Cheng, Jihua Meng, Jiali Shang, Jiangui Liu, Jianxi Huang, Yanyou Qiao, Budong Qian, Qi Jing, Taifeng Dong, Lihong Yu

**Affiliations:** 1 Institute of Geography, Fujian Normal University, Fuzhou 350007, China; chengzq@fjnu.edu.cn; 2 Key Laboratory of Digital Earth, Aerospace Information Research Institute, Chinese Academy of Sciences, Beijing 100101, China; qiaoyy@radi.ac.cn; 3 Key Laboratory of Humid Subtropical Eco-Geographical Process (Fujian Normal University), Ministry of Education, Fuzhou 350007, China; 4 Ottawa Research and Development Centre, Agriculture and Agri-Food Canada, Ottawa, ON K1A 0C6, Canada; Jiali.Shang@canada.ca (J.S.); Jiangui.Liu@canada.ca (J.L.); Budong.Qian@canada.ca (B.Q.); Qi.Jing@canada.ca (Q.J.); dong.taifeng@gmail.com (T.D.); 5 College of Land Science and Technology, China Agricultural University, Beijing 100094, China; jxhuang@cau.edu.cn; 6 Faculty of Geographical Science, Beijing Normal University, Beijing 100875, China; Yulh@mail.bnu.edu.cn

**Keywords:** crop growth, reflectance saturation, crop model, assimilation, crop growth stage, method combinations

## Abstract

Green leaf area index (LAI) is an important variable related to crop growth. Accurate and timely information on LAI is essential for developing suitable field management strategies to mitigate risk and boost yield. Several remote sensing (RS) based methods have been recently developed to estimate LAI at the regional scale. However, the performance of these methods tends to be affected by the quality of RS data, especially when time-series LAI are required. For crop LAI estimation, supplementary growth information from crop model is helpful to address this issue. In this study, we focus on the regional-scale LAI estimations of spring maize for the entire growth season. Using time-series multispectral RS data acquired by an unmanned aerial vehicle (UAV) and the World Food Studies (WOFOST) crop model, three methods were applied at different crop growth stages: empirical method using vegetation index (VI), data assimilation method and hybrid method. The VI-based method and assimilation method were used to generate time-series LAI estimations for the whole crop growth season. Then, a hybrid method specially for the late-stage LAI retrieval was developed by integrating WOFOST model and data assimilation. Using field-collected LAI data in Hongxing Farm in 2014, the performances of these three methods were evaluated. At the early stage, the VI-based method (R^2^ = 0.63, RMSE = 0.16, *n* = 36) achieved higher accuracy than the assimilation method (R^2^ = 0.54, RMSE = 0.52, *n* = 36), whereas at the mid stage, the assimilation method (R^2^ = 0.63, RMSE = 0.46, *n* = 28) showed higher accuracy than the VI-based method (R^2^ = 0.41, RMSE = 0.51, *n* = 28). At the late stage, the hybrid method yielded the highest accuracy (R^2^ = 0.63, RMSE = 0.46, *n* = 29), compared with the VI-based method (R^2^ = 0.19, RMSE = 0.43, *n* = 28) and the assimilation method (R^2^ = 0.20, RMSE = 0.44, *n* = 29). Based on the results above, we considered a combination of the three methods, i.e., the VI-based method for the early stage, the assimilation method for the mid stage, and the hybrid method for the late stage, as an ideal strategy for spring-maize LAI estimation for the entire growth season of 2014 in Hongxing Farm, and the accuracy of the combined method over the whole growth season is higher than that of any single method.

## 1. Introduction

Leaf area index (LAI) is defined as the one-sided green leaf area per unit ground area in broadleaf canopies and as the projected needle leaf area in coniferous canopies [1,2]. Because green LAI (LAI_g_) determines light interception and absorption of the crop canopy [3], estimation of crop LAI is critical for understanding the biophysical processes of crop growth that are essential for predicting crop biomass or yield [4,5].

To achieve accurate LAI estimations, a number of remote sensing (RS) based approaches have been developed over the past few decades. These approaches can be grouped into two broad categories: empirical methods and physical models [6]. Empirical methods can be further divided into parametric and non-parametric empirical methods based on whether there is an explicit relationship between selected RS bands and LAI [7]. A series of optical vegetation indices (VIs) have been developed through combinations of reflectance in two or more bands [8,9] for maize LAI estimation. For instance, a normalized difference vegetation index (NDVI) [10] calculated from the reflectance in red and near-infrared bands are commonly used for retrieving canopy biophysical properties of corn [11]. Because NDVI is highly affected by soil reflectance at low LAI and shows asymptotic saturation at high LAI [12], some more VIs were designed, such as the optimized soil adjusted vegetation index (OSAVI) [13] for considering the soil effect and the two-band enhanced vegetation index (EVI2) [14] for tackling reflectance saturation. The building of empirical methods depends on ground LAI observations which were generally made using different instruments [15,16,17]. Using ground LAI measurements and RS-based VIs, several empirical methods have been developed for maize LAI estimations [7,10,11]. The non-parametric empirical methods usually define the regression function between the RS information and the target variable, e.g., LAI directly [18]. Machine learning techniques, include neural network [19], support vector regression [20] and gaussian processes [21], are typical non-parametric empirical methods to generate LAI products from a variety of RS data products. Although empirical methods are effective in estimating LAI with high accuracy, they are site- and time-specific [22].

Physical models are constructed based on simulations of radiative transfer process in vegetation canopy, and therefore their utilities are not limited to a specific site or a certain time of a year as empirical methods. Physical models can be further divided into geometric optical (GO) model [23], radiative transfer (RT) model [24] and RT-GO model [25]. In these models, LAI is one of the canopy parameters that determine canopy reflectance. With the canopy reflectance acquired from RS data and other canopy parameters known, these models can be inverted to derive LAI. RT models are more suitable for crop LAI retrievals than GO models as a result, the PROSAIL model, a RT model, has been commonly used to simulate LAI for maize using RS data [26,27]. The limitations of physical models include the high cost of parameters calibration and low model running efficiency. Recently, a physical model and a machine learning technique were combined for LAI estimations [6]. For instance, the PROSAIL model and a neural network algorithm were combined to estimate maize LAI with high accuracy [28]. The machine learning algorithm can help improve the efficiency of physical models, but the high cost of parameters calibration cannot be completely avoided.

Because crop growth is a long-term process, continuous LAI information for the entire growth season is critical to calculate corresponding biomass at different growth stages and thus essential to predict the final yield. Since empirical methods and physical models require RS data as inputs for LAI estimation, the quality of RS data will influence the estimation accuracy, especially when time-series RS data are required to generate LAI estimations for different crop growth stages. In the rainy season in northeast China, clouds, shadows, and haze can strongly influence the application of multispectral RS data with high and medium spatial resolutions in time-series LAI estimation. Because plants usually develop fast in the rainy reason, the lack of good quality RS data will cause the LAI estimates to miss the critical growth stage, and thus will consequently influence biomass or yield estimation. At the late-growth stage, the appearance of more senescing leaves will limit the RS application of LAI estimations [29]. The senescing leaves are still attached to plants and can be captured by in-situ LAI instruments. However, these leaves have reduced photosynthetic capacity and therefore present quite difference spectral behaviors from that of green leaves. The presence of senescing leaves can be at different extents at the late stage [14,30], which is a great source of uncertainty for canopy LAI estimation from RS data. The saturation of reflectance and VIs is another significant limitation for the late-growth stage. The EVI has been designed to provide improved sensitivity at high LAI [31]; however, it is still not sufficient for the spring maize.

As mentioned above, the quality issues of RS data may cause failures of directly applying RS-based empirical and physical models for LAI estimation in rainy seasons or at the late-growth stage. Hence, supplementary information should be referred to address this issue. Unlike other vegetation types, crops usually own a more regular growth period which can be useful in building mechanistic models for simulating crop growth. Crop models [32,33] are able to provide comprehensive mathematical descriptions of key crop physical and physiological processes, which cannot be achieved by RS-based methods. Therefore, it is more applicable to use crop models for LAI estimations during rainy- and late-growth seasons.

Since crop models are generally designed to simulate crop growth at the site level, several data assimilation approaches [34,35,36] have been developed to incorporate crop parameters estimated from RS data into crop model, aiming to extrapolate the simulations of crop models from a single site to the regional scale. Among them, the Ensemble Kalman Filter (EnKF) [37,38,39], particle filtering (PF) [40], and four-dimensional variational data assimilation (4DVar) [41,42] are common methods used to link crop models with RS data. Unlike RS-based LAI estimation methods, assimilation methods can tolerate time-series RS data withlong time intervals, making it possible to retrieve accurate LAI estiamtions in rainy season. Therefore, it is feasible to use crop models to fill data gaps where high-quality continuous RS data are not available. Because assimimation methods still require a small amount of RS data, it cannot address the quality issues of RS data at thelate-growth stage. Hence, further studies are needed to improve the multi-phase LAI estimates by integrating RS data and crop models.

The objective of this study is to address the quality issues of RS data for time-series LAI generation. Using the World Food Studies (WOFOST) crop model and RS data acquired by an unmanned aerial vehicle (UAV) in 2014, three methods were applied to estimate LAI for spring maize in Hongxing Farm. Based on the performances of these methods, a method combination was developed to generate LAI for the entire growth season, which includes: (1) a VI-based empirical method for early-stage LAI estimation before rainy season; (2) the EnKF assimilation method for rainy-season LAI generation; (3) the hybrid method by integrating EnKF method and WOFOST model for late-stage LAI retrieval. Complete details of the applied methods and their performances were presented in the following sections.

## 2. Materials and Methods

### 2.1. Study Area and Field Campaign

**Figure 1:**
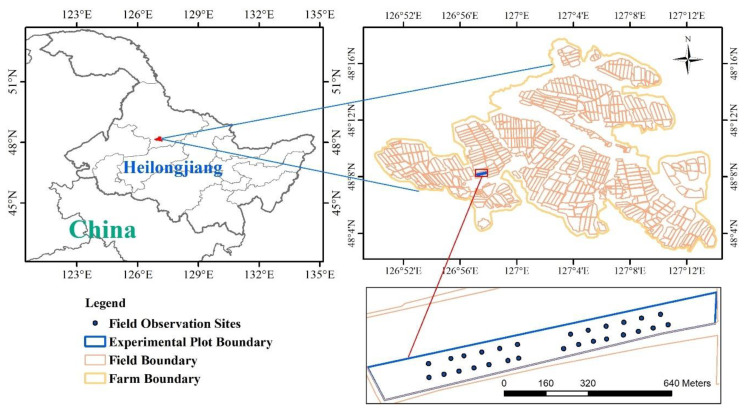
Locations of the study area and the field observation sites.

The study was conducted in an experimental field (48°08′ N, 126°57′ E, WGS84) of Hongxing Farm, located in Heilongjiang province, Northeast China. Hongxing Farm is a large state-owned farm that lies within the temperate monsoon climate zone characterized by an average annual precipitation of 548.8 mm (2014) and an average annual cumulative temperature (>10 °C) of 2293.0 °C (2014). Spring maize accounts for nearly 50% of the planting area of Hongxing Farm. The spring maize growing season extends from the beginning of May till mid-October. Another important crop in this region is soybean, which is usually rotated with spring maize. All the fields in Hongxing Farm have a unique identifier (ID). The ID of the experimental field selected for this study is 5-1-2. Plot 5-1-2 covers 11 hectares (ha), and seeded with spring maize on 18 May 2015. Figure 1 shows the location of the study area and the spatial distribution of field observation sites.

A total of six field campaigns were conducted in 2015 to collect data for algorithm development. During the field campaigns, LAI, basic soil available nutrient (SAN) contents, yield, and growth period were observed. For LAI, three sequential field campaigns were conducted on 29 June, 30 July and 25 August. The number of quadrats involved in these campaigns were 36, 28, and 29 respectively. The isometric sampling method was used to establish these quadrats. The distance of each quadrat is more than 100 m and the locations for 29 June are shown in Figure 1. The area of each quadrat was 4 m × 6 m. The LAI-2000 Plant Canopy Analyzer (LI-COR) [15,43] was used to measure LAI of each quadrat. Using an optical sensor, the LAI-2000 can measure the effective LAI, under the assumption of random leaf spatial distribution. Besides LAI, the instrument can also calculate the mean foliage inclination and fraction of the sky visible from beneath the canopy. In this study, a one-up-seven-down scheme was used to measure the LAI, i.e., in each quadrat, we obtained one measurement of sky light above the canopy and then seven measurements of diffuse light below the canopy. The final LAI was calculated using an LAI-2000 analyzer. Visually recognizable brown leaves in the target quadrats were eliminated before measurements so that in-situ LAI measurements only accounts for green leaves and thus can be comparable with LAI from optical RS data.

Another field campaign was conducted to collect basic SAN contents at the same locations and with the same quadrat size as the LAI campaigns, on 10 May 2015 before the application of basic fertilizer. In each quadrat, three sampling points along the diagonal were located, from which soil samples were collected. The collected samples were used to measure the available nitrogen (N), phosphorus (P), and potassium (K) contents in the lab. The mean value of the three sampling points was calculated and used as the basic SAN content of each quadrat. For crop yield, we collected the total grain weight of the plot after harvesting. The total grain weight divided by experimental plot area is used as the measured yield. The percentage impurity and water content were also recorded, and the final yield was calculated by removing the impurity and adjusting for the water content at 25%.

The key growth stages were also observed and detailed information on LAI, SAN, yield and growth stage experiments, including the sampling strategies, soil sampling procedures, and testing methods, can be found in [44,45].

### 2.2. Remote Sensing Data Acquired by the Unmanned Aerial System (UAS)

**Figure 2:**
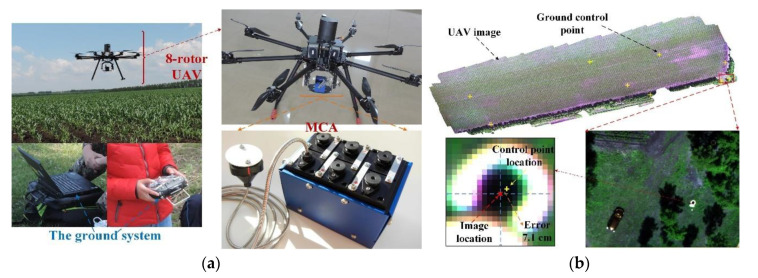
The components of the UAV platform (**a**) and geometric correction accuracy (**b**).

The RS data used in this study were acquired by a UAS. The UAS consists of three parts: an 8-rotor UAV, an MCA (Multispectral Camera Array) system, and a ground system, as shown in Figure 2. The MCA camera acquires five-band image with a resolution of 1280 × 1024 (1.3 M) pixels. The central wavelengths of the five bands are 490 nm (blue), 550 nm (green), 680 nm (red), 720 nm (red edge) and 800 nm (near-infrared). The flight height was set to 100 m above the ground level, and the overlap for the flight lines was 60% longitudinally and 40% laterally.

The image pre-processing includes reflectance calculation, image stitching, and geometric correction. Among the 6 MCA channels, one of them was connected to an electronic component (an e-ILS sensor) to receive incoming radiation. Using the incident solar radiation, the reflectance for the other five channels was obtained, thus the reflectance can be called at-sensor-reflectance. Because the UAV flight was conducted under clear weather condition and the fight height is 100 m, the at-sensor-reflectance can be used as the reflectance of crop canopy without the need for atmospheric correction in this study. Two software, the Agisoft PhotoScan and PIE-UAV (Pixel Information Expert), were used to generate the ALS-similar point clouds and to stitch the images using information on the flight height, camera attitude, and GPS. For geometric correction, 18 ground control points were collected within the experimental field using a hand-held Trimble GeoXH differential GPS with a mean estimated error of 0.10 m. Twelve points were used to make the geometric correction and six points were used to calculate the mean deviation error (MDE) of the corrected UAV images. The results show that the MDE was less than two pixels (0.1 m) for the June and July images and three pixels (0.15 m) for the August image. After reflectance calculation, image stitching and geometric correction, six images with a spatial resolution of 0.054 m × 0.054 m were obtained.

### 2.3. Estimation of LAI

**Figure 3:**
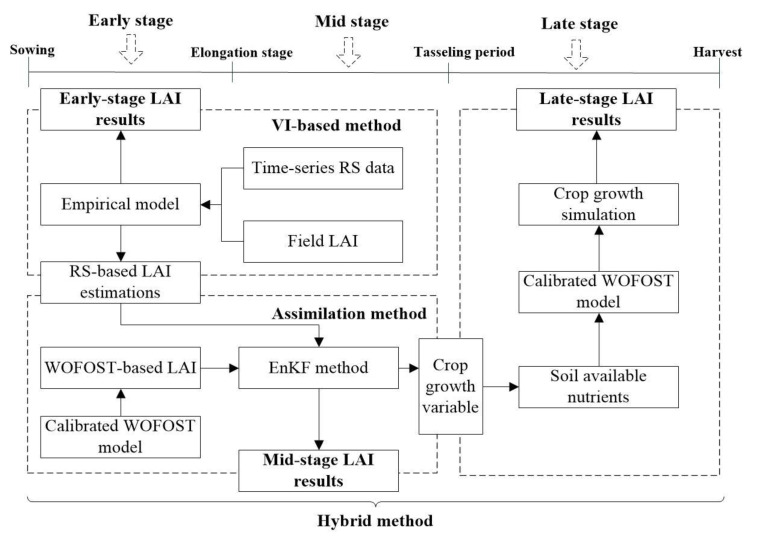
Processes of LAI estimation for the entire growth season using combined methods.

The quality issues of RS data will cause failures of common RS-based empirical methods and physical models for continuous LAI estimations for the entire growth season. Hence, crop models were considered in this study to avoid the use of low quality RS data. By integrating the time-series RS data acquired by the UAS and WOFOST crop growth model, the LAI was estimated using combined methods and the processes of LAI estimation for the entire growth is shown in Figure 3.

In Figure 3, the whole growth season was divided into three stages: the early stage, the mid stage, and the late stage. The phase from the emergence to the end of the elongation stage was considered as the early stage, and the beginning of the late stage is the tasseling period in this study. The mid stage was defined as the phase between the early and the late stage. In general, the early stage includes the period with high-quality RS time series, the mid stage can also be considered as the rainy season in this study before leaf senescence, and the late stage are characterized by reflectance saturation and leaf senescence. Based on field observations, we divided the specific time spans for the early stage (from the beginning of the growing season to 14 July) and late stage (from 12 August to the end of the growth season). The 14 July and 12 August dates were determined by calculating the middle dates of the field campaigns (30 June, 29 July and 25 August). Three methods, including the VI-based method, the assimilation method, and the hybrid method integrating crop model and the assimilation method, were applied to estimate LAI for the three stages, respectively. The details of these methods are in Section 2.3.1, Section 2.3.2 and Section 2.3.3.

#### 2.3.1. VI-Based Method

Our previous study [44] showed that the empirical model can provide LAI estimates with higher accuracy than that of the physically based model (e.g., via PROSAIL simulation). In this study, the relationship between different VIs and ground LAI were analyzed, and the VI with the highest accuracy was selected to build a linear empirical model to estimate LAI.

To ensure the accuracy of LAI estimation, five VIs were calculated from the UAV data. Besides the commonly used *NDVI* and ratio vegetation index (*RVI*) [8], *OSAVI* was also selected to reduce the effect of soil at low LAI, and *EVI2* and modified triangular vegetation index (*MTVI2*) [9] to lower the influence of reflectance saturation. The selected VIs were calculated using the following equations:

(1) $$NDVI=\frac{NIR-RED}{NIR+RED}$$

(2) $$RVI=\frac{NIR}{RED}$$

(3) $$OSAVI=\frac{1.16\times \left(NIR-RED\right)}{NIR+RED+0.16}$$

(4) $$EVI2=\frac{2.5\times \left(NIR-RED\right)}{NIR+2.4\times RED+1}$$

(5) $$MTVI2=\frac{1.5\times \left\{1.2*\left(NIR-GREEN\right)-2.5\times \left(RED-GREEN\right)\right\}}{\sqrt{{\left(2\times NIR+1\right)}^{2}-\left(6*NIR-5\times \sqrt{RED}\right)-0.5}}$$

where *NIR*, *RED* and *GREEN* denote the reflectance of the near-infrared band, red band and green band, respectively. Based on the calculated VIs and ground LAI, linear functions can be built. The VI yielding the highest accuracy of LAI estimation was selected as the optimal method for the early-stage LAI estimation.

#### 2.3.2. Data Assimilation Method

As shown in Figure 3, the WOFOST model and EnKF method was used to apply RS assimilation for mid-stage LAI simulation. As a primary member of the Wageningen crop models [46] and a core component of the Crop Growth Monitoring System (CGMS) [47], the WOFOST model can simulate daily crop physiological and ecological processes. There were two reasons to select the WOFOST model. Firstly, it can predict daily LAI by simulating CO_2_ assimilation, respiration, leaf growth and dry matter formation. Secondly, following model modifications referred in [44], WOFOST can simulate LAI under nutrient-limited conditions. Compared with the water-limited LAI, the accuracy of estimated nutrient-limited LAI can be improved by eliminating the influence of SAN on crop growth.

Before WOFOST can be used for LAI simulation, its main parameters must be calibrated. In this study, three methods were used to acquire the input parameters: the SAN estimation method, field campaigns, and the FSEOPT optimization software. The method proposed in [44] was used to estimate the SAN content. Field data was also used to calibrate crop and soil water parameters. Details of the field campaign method can be found in [45]. However, there are still some core parameters cannot be calibrated using the above two methods. In this study, the FSEOPT optimization software [48] was used to calibrate the uncalibrated parameters based on field LAI observations. We selected LAI because it is the research subject of this study. The mean field LAI value of the experimental plot was calculated as the output variable. Then the calculated LAI was used to conduct optimization in the FSEOPT software. The optimization was conducted once using LAI data collected on each date of 30 June, 29 July and 25 August, respectively. Three values for each parameter were acquired after three-time FSEOPT performances and the mean value was calculated as the final calibrated parameter. The calibrated WOFOST model was used to simulate daily crop growth and output LAI results, which is necessary for the assimilation method.

In this study, the EnKF method [37] was adopted to assimilate the LAI estimated from the VI-based method into the WOFOST model to correct for daily simulated LAI results, and LAI values after data assimilation were taken as the output results for the mid stage. The EnKF method, which is based on the Monte Carlo simulation, performs a model forecast in which the state variables are propagated forward in time based on the modeled dynamics and updated using probability distribution and available observations [49]. EnKF is a major assimilation method that can be easily applied in the WOFOST model [50]. In the recursive algorithm of the Kalman Filter, the assimilation process is divided into two steps [41]: forecast and update. In the forecast step, the covariance matrix (*A_c_*) of the state variables is calculated. If ensemble of the state variables is defined as *A_t_*,

(6) $$A_{t}=\left(x_{1},x_{2},x_{3},\ldots ,x_{N}\right)$$

then *A_c_* can be calculated as follow:

$$A_{c}=\left(A_{t}-\bar{A}\right)*{\left(A_{t}-\bar{A}\right)}^{T}/\left(N-1\right)$$

where $\bar{A}$ is the mean value of *A_t_*, *T* represents the transpose of a vector, *N* is the element number in *A_t_*.

In the update step, the state variables were updated using the observation (RS based LAI). The simulated ensemble of state variables was calculated by a nonlinear equation:

(7) $$x_{i}\left(t\right)=M\left[x_{i}\left(t-1\right)\right]$$

where *t* is WOFOST simulation step (Day of year), *M* is the forecast equation.

If the observed ensemble (RS based LAI) was defined as *D_t_*, the covariance matrix (*D_c_*) of the observed ensemble can also be calculated by Equation (6). Then the standard analysis equation can be built to calculate the updated WOFOST simulated ensemble (*A_a_*):

(8) $$A_{a}=A_{f}+K\left(D_{t}-HA\right)$$

where *A_a_* is the optimal estimated ensemble, *A_f_* is the forecasted ensemble calculated using Equation (7), *K* is the Kalman gain matrix to weigh the difference between observation (RS-based LAI) and prior simulation of the model’s state (WOFOST-based LAI), which was calculated by the following equation:

(9) $$K=A_{c}H^{T}{\left(HA_{c}H^{T}+D_{c}\right)}^{-1}$$

where *H* is the parameter of observation operator. The *A_a_* was used to replace *A_t_* to realize the assimilation process.

Filter divergence was observed in our study. It represents a tendency of the standard EnKF to reject observations in favor of ensemble forecasting in subsequent stages, leading to incremental deviations of LAI from actual measurements. To address this issue, an expansion parameter (*E*) was used:

(10) $$E=R*N*\frac{\sigma_{1}^{2}}{D*\sigma_{2}^{2}}$$

where *R* is a random number that is less than 1, *D* is the total number of days for the assimilation stage, *N* is the number of days (from 1 to *D*) for the current WOFOST’s simulation step, $\sigma_{1}^{2}$ is the variance of RS based LAI of *N*, and $\sigma_{2}^{2}$ is the variance of WOFOST-based LAI of *N*. When $\sigma_{1}^{2}/\sigma_{2}^{2}$ is larger than 4 and *E* is equal or greater than 1, E will be used to enlarge *K* to eliminate filter divergence in this study.

#### 2.3.3. The Hybrid Method

**Figure 4:**
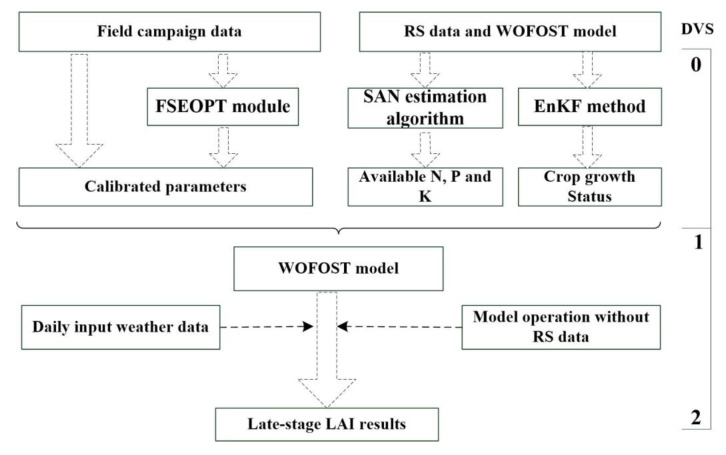
Processes of LAI estimation for the late stage using hybrid method (DVS: the development stage of the crop, DVS = 1 indicates the beginning of tasseling period for maize in this study).

As mentioned in the introduction, both VI-based and assimilation methods cannot address the quality issues of RS data at the late-growth stage caused by senescing leaves and reflectance saturation. Hence, we designed a new strategy (the hybrid method) for the late-stage LAI estimation. In the hybrid method, the WOFOST model with and without UAV data assimilation were combined to improve the late-stage LAI estimation. The UAV data were assimilated into the WOFOST model to generate daily crop growth from the beginning of the growth season until the begin of late stage. Then the data assimilation was halted, and WOFOST was only used to simulate LAI until the end of the growth season. The processes of LAI estimation for the late stage is shown in Figure 4.

Figure 4 indicates that the hybrid method can be considered as a specific crop model method in which the WOFOST model without using RS data was run from the end of the mid stage (DVS = 1). To enable the simulation of WOFOST, the parameters of crop growth status with DVS = 1 and RS-based SAN contents were required as initial inputs. Then, the WOFOST model was self-operated and daily LAI values were output as the late-stage results. This method was named as the hybrid method to distinguish it from the WOFOST model used in the assimilation method.

Finally, three methods were combined to generate LAI time series for the entire growth season (shown in Figure 3). Detailed LAI estimation processes are described as follows:

Step 1. For early-stage LAI estimation, the VI-based empirical method was applied. VIs were calculated from time-series RS data acquired by UAS and the empirical model was built between field LAI measurements and VIs. Then, LAI was estimated using the empirical model.
Step 2. For mid-stage LAI estimation, the RS-based LAI was assimilated into the WOFOST model to correct the daily crop growth simulations from the beginning of the growth season to the end of the mid stage to generate LAI estimations.
Step 3. For late-stage LAI simulation, crop growth was simulated using the method in Step 2 from the beginning of the growing season to the end of the mid stage. Then, we halted RS assimilation and used the WOFOST model instead to output the daily LAI.

### 2.4. Evaluating the Accuracies of LAI Estimations

The performance of the three LAI estimation methods was evaluated using field LAI data. The correlation coefficient (*R*), coefficient of determination (*R*^2^) and *RMSE* were selected as the indices to analyze the relationship between the estimated and field-measured LAI values. The *R*^2^ and *RMSE* were calculated as follows:

(11) $$L_{mean}=\frac{1}{n}\overset{n}{\underset{k=1}{\sum }}L_{obs,k}$$

(12) $$L_{f}=a\times L+b$$

(13) $$R^{2}=\frac{\sum_{k=1}^{n}{\left(L_{f,k}-L_{mean}\right)}^{2}}{\sum_{k=1}^{n}{\left(L_{obs,k}-L_{mean}\right)}^{2}}$$

(14) $$RMSE=\sqrt{\frac{\sum_{k=1}^{n}{\left(L_{k}-L_{obs,k}\right)}^{2}}{n}}$$

where *L_obs_* is the measured LAI values during the first three field campaigns introduced above, *n* is the number of quadrats, *L_mean_* is the mean value of *L_obs_*, *L* is the RS-based VIs (vegetation index method) or simulated LAI (WOFOST model and assimilation method), *L_f_* is the linear fitting formula between *L* (independent variable) and *L_obs_* (dependent variable), *a* and *b* are the coefficients.

The *R*^2^ and *RMSE* were both used to evaluate the performance of LAI estimation methods. Generally, the LAI estimation method with a higher *R*^2^ corresponding to a lower *RMSE* was selected as the ideal method combinations for time-series LAI estimation.

Furthermore, the variable coefficient (*CV*) was selected to examine the spatial heterogeneity of late-stage LAI estimations. The *CV* was calculated as follows:

(15) $$CV=\frac{SD}{Mean}*100\%$$

where *SD* is the standard deviation and *Mean* is the mean value of the research data (LAI and vegetation indices in this study).

## 3. Results and Discussion

### 3.1. LAI Estimation Using the VI-Based Method

Using the linear regression model, the R values between RS-based VIs and field LAI were derived. To show the significant correlation level, a significance test was also conducted by calculating the *p*-value. *p*-Value < 0.01 indicates highly significant correlation level and *p*-value < 0.05 for significant correlation level. The R values and significance levels are listed in Table 1. The results show that RVI provided the best LAI estimations with the highest R among all the indices on 30 June (the early-growth stage) and 29 July (the mid-growth stage). EVI2 provided LAI estimate with the highest R on 25 August (the late-growth stage). The R values on 30 June (RVI) and 29 July (RVI) reach highly significant level, reaches significant level on 25 August (EVI2).

**Figure 5:**
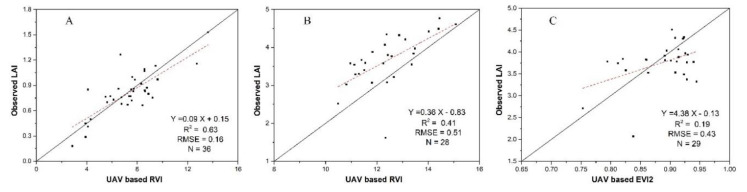
Established empirical vegetation index models (**A**) 30 June, (**B**) 29 July, (**C**) 25 August.

Subsequently, RVI and EVI2 were selected to build the RS-based statistical models for LAI estimation. The R^2^ and RMSE values of the field LAI and estimated LAI were also calculated. The statistical models and the accuracy analysis results are shown in Figure 5.

From Figure 5, we can observe that the estimation accuracy on 25 August was considerably lower than that on 30 June and 29 July. The VI saturation that occurs during the late-growth season is an important reason for the significantly reduced accuracy on 25 August. In Table 2, the CV indices of the five VIs on 30 June, 29 July and 25 August were calculated to represent the levels of VI saturation on different dates. The results (listed in Table 2) show that the CV values on 29 July and 25 August were lower than that on 30 June, which means that there is a saturation trend toward the mid and late season.

**Figure 6:**
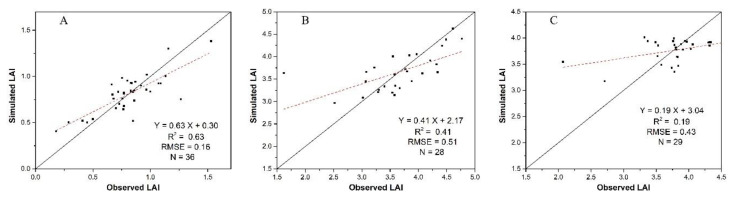
The accuracy of LAI estimations using the vegetation index method (**A**) 30 June, (**B**) 29 July, (**C**) 25 August.

To compare the levels of VI saturation at different stages, LAI estimations using the VI-based method were shown in Figure 6. The figure reveals that the relative dynamic range (the dynamic range ratio of simulated and observed LAI) was reduced on 25 August respective to 29 July and 30 June. This result suggests that although the saturation has already appeared on 30 July, it is more obvious on 25 August. Hence, the saturation is an important reason for the poor performance of VI-based empirical method for LAI estimation at the late stage.

Besides VI saturation, leaf senesce may also have contributed to the lower estimation accuracy on 25 August. At the late growth stage of spring maize, the primary receiver of dry matter from photosynthesis will be shifted to storge organs. The WOFOST’ parameters FOTB (fraction of above ground dry matter to storage organs) and FLTB (fraction of above ground dry matter to leaves) can be used to demonstrate this phenomenon. The FOTB on 25 August is higher than that on 29 July, while FLTB shows the opposite pattern (25 August: FOTB = 0.69, FLTB = 0.12; 29 July FOTB = 0.20, FLTB = 0.18). Less dry matter supply will decrease leaf bioactivity and thus generate more inactive leaves. Chemical changes in inactive leaves can hardly be detected by optical RS data. In general, saturation and senescence jointly decreased the correlation between LAI and RS-based VIs.

From Figure 6A, we can find that the accuracy of VI-based method for the early-stage LAI estimation is higher than the assimilation method. Hence, the statistical model shown in Figure 5A was selected as the ideal method to provide early-stage LAI results (shown in Figure 3). Furthermore, the RS-based LAI was also required by the assimilation method, and the three statistical models shown in Figure 5 were used to generate the RS-based LAI for the entire growth season. Then the inverse distance weighted interpolation method was used to generate daily RS-based LAI for data assimilation.

Although the VI-based statistical models can provide higher accuracy LAI estimations, these models are commonly used for specific sensors and sampling conditions because the lack of physical basis. Thus, any changes in location, crop type and application years can cause failures in the original model application [51,52]. Because radiative transfer model [53] is the core algorithm of the physical models, they reflect the transfer and interaction of radiation inside the canopy; hence, the physical model methods can overcome the limitations of sensors, geographical locations, and application times. A physical model [26] should be considered before applying the LAI estimation method to a larger area with different crops for different years. Meanwhile, detailed radiation transfer and interaction simulations in physical models require more input parameters; consequently, more field experiments should be conducted before applying physical models. Because there is no significant change in environmental conditions, growth stages, and remote sensing data calibration, the VI-based statistical model was selected and re-calibration of the relationship between RS-based VI and field LAI each time is not necessary.

### 3.2. LAI Simulation Using the Assimilation Method

#### 3.2.1. WOFOST Model Calibration

The results of the WOFOST model calibration are presented in this section. The input parameters of the WOFOST model include meteorological, soil, and crop parameters. In addition to the daily meteorological and fertilization data, which were provided by Hongxing Farm, other parameters also need to be calibrated in this study. Based on a sensitivity analysis (discussed in previous work [44]), 18 parameters sensitive to LAI were selected as core parameters to be calibrated using the three previously described calibration methods in this study. The values of the calibrated parameters and their calibration methods are listed in Table 3.

The accuracy of simulated crop growth by the calibrated WOFOST model was firstly evaluated using emergence time, anthesis time, maturity time and yield. These parameters were simulated for the experimental plot and compared with field observations, and the results are listed in Table 4. Comparing to observations, the crop growth, as indicated by those parameters in Table 4, simulated by the calibrated model is better than the original model.

#### 3.2.2. LAI Simulation Using the WOFOST Model

**Figure 7:**
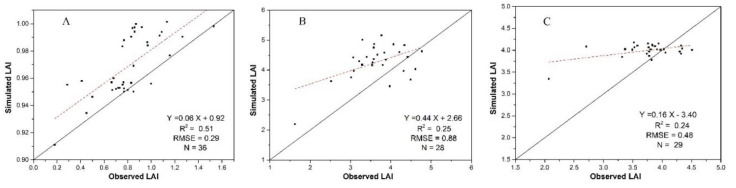
The LAI estimation accuracies for the calibrated WOFOST model (**A**) 30 June, (**B**) 29 July, (**C**) 25 August.

We applied the calibrated WOFOST model to simulate LAI which is required for the assimilation process, and the model performances were also evaluated using field LAI. Results (shown in Figure 7) show that the calibrated model can simulate LAI on 30 June with high accuracy, but yielded LAI estimates with much lower accuracy on 25 July and 25 August. Calibration errors are the potential reason for the lower accuracy at the mid- and late-stages. Some core parameters of the WOFOST model can only be acquired as constant values at the site level; but in reality they may change with time. Thus, errors cannot be completely avoided using either of the three calibration methods. These errors will propagate and accumulate, hence reduce the accuracy of LAI simulation over time. The higher simulation accuracy on 30 June than the other two dates also indicates that reducing the simulation time can weaken the calibration error propagation and accumulation and help the WOFOST model provide better LAI simulation.

**Figure 8:**
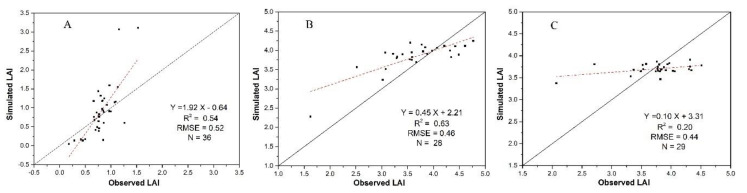
The LAI estimation accuracies using assimilation method (**A**) 30 June, (**B**) 29 July, (**C**) 25 August.

Although the single WOFOST model cannot provide LAI estimates with higher accuracy than the VI-based method and the assimilation method for all the three growth stages (shown in Figure 6, Figure 7 and Figure 8), WOFOST was still required by the assimilation method and the hybrid method (shown in Figure 3). Errors in the WOFOST-based growth simulation will be reflected in the performance of the assimilation and the hybrid methods. Hence, improve the performance of WOFOST is beneficial for the estimation of LAI time series in this study. Because LAI is simulated and analyzed at the pixel or sampling point level, the parameters with high spatial heterogeneity should also be calibrated at the pixel level to ensure the LAI simulation accuracy. Among input parameters, soil parameters, including soil water and soil available nutrients, are the main variables that change within the experimental field in this study. Considering that soil water contents can be simulated by WOFOST model using daily precipitation data, the SAN values at the pixel level should be acquired before conducting WOFOST-based LAI simulation.

The SAN estimation method proposed in [44] was used to provide available N, P and K based on RS data and the WOFOST model. From the accuracy analysis results, we can find that the estimation accuracy for K (R^2^ = 0.15, RMSE = 23.56 mg/kg, mean = 168.26 mg/kg) was significantly lower than that of N (R^2^ = 0.48, RMSE = 18.45 mg/kg, mean value = 296.78 mg/kg) and P (R^2^ = 0.37, RMSE = 7.05 mg/kg, mean value = 31.63 mg/kg). Optimizing the K estimation would be useful to further improve the LAI simulations. The instability of K and the lower effect of potassium ions in comparison with the other two ions on crop growth, especially on leaf growth, are the possible causes for the lower estimation accuracy using the proposed method. Adjusting the SAN estimation method to consider these factors will be the focus of future studies to further improve the K estimation accuracy.

#### 3.2.3. LAI Simulation Using the EnKF Assimilation Method

Using the method presented in Section 3.1, LAI was estimated using time-series UAV data first, and then assimilated into the WOFOST model through the EnKF method. The calibrated model with data assimilation was used to generate LAI for 30 June, 29 July and 25 August. The R^2^ and RMSE were calculated for the three LAI simulations using field LAI. The results (listed in Figure 8) show that the UAV data improved the WOFOST model’s LAI simulation performance for 30 June and 29 July. However, the accuracy for 25 August did not improve. Compared with the VI-based empirical method, the assimilation method provides higher simulation accuracy for 29 July and similar accuracies for 30 June and 25 August. From Figure 8A, we see that there are two samples with higher simulated LAI (around 3). Comparing Figure 6B and Figure 8B, we can also find that the assimilation can provide mid-stage LAI estimation with higher accuracy, thus, this method was selected as the ideal method for this stage (shown in Figure 3).

**Figure 9:**
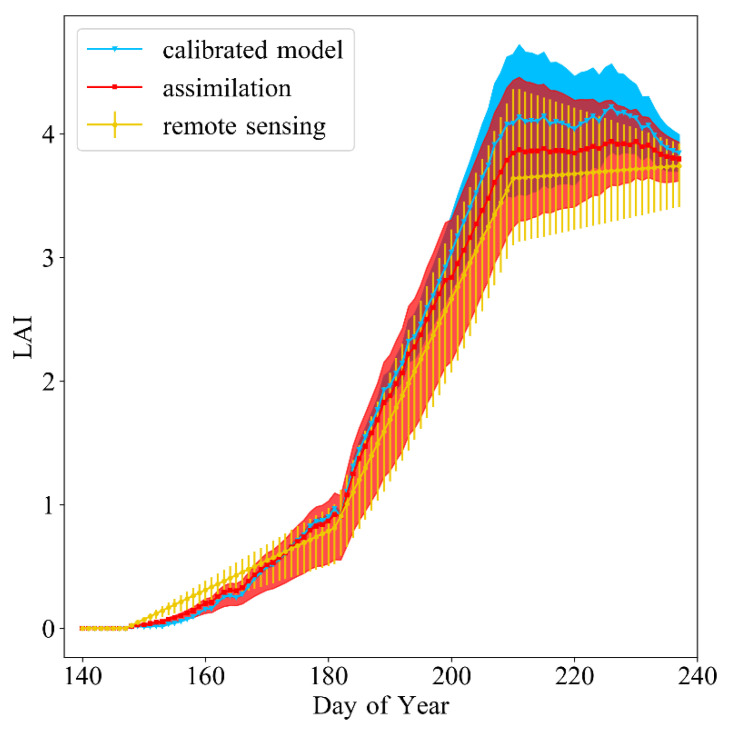
The seasonal LAI estimated using three methods: the calibrated WOFOST model, the assimilation method, and the VI-based empirical using RS data.

To show the improvements that both calibration and assimilation can bring in LAI simulation, the LAI trajectories were gathered from three methods including the calibrated WOFOST model, the assimilation method, and the VI-based empirical method. We selected the mean LAI value of the experimental plot and LAI’s variation range (10–90%) as the indexes and generate three trajectories (shown in Figure 9). The results show that the assimilation can correct WOFOST’s LAI simulation to certain extent. Because we didn’t conduct UAV flight after 25 August, the trajectories end at that time (Day of year; DOY = 137).

### 3.3. LAI Estimations Using the Hybrid Method

**Figure 10:**
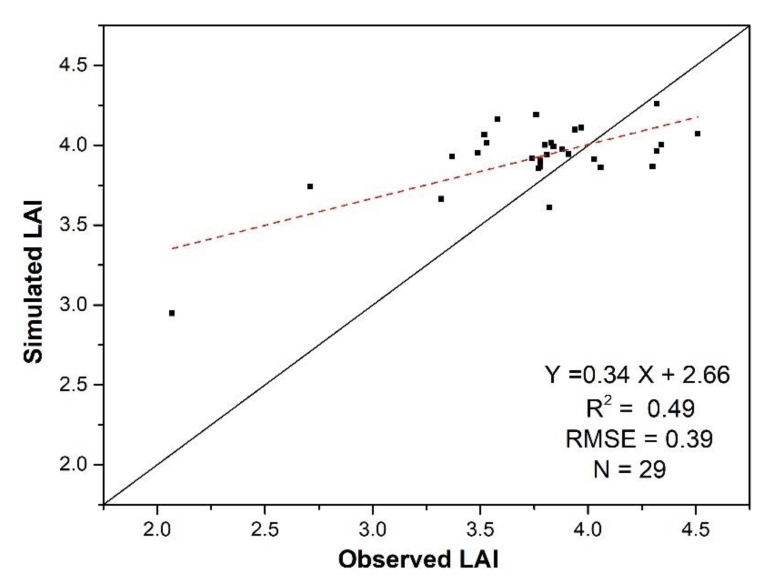
The LAI estimation accuracies for 25 August using the hybrid method.

From above analyses, we can find that the late-stage LAI estimation accuracies of the VI-based, the WOFOST model and the assimilation methods at the late stage are much lower than those at the early and mid-stages. Model calibration, reflectance and VI saturation, and canopy changes associated with leaf aging, jointly contributed to the reduced performance to certain extents. Hence, the hybrid method was developed to improve the accuracy of AI estimation at the late stage. Using this method, LAI values on 25 August were simulated, and the accuracy was evaluated using field LAI. The analysis result, which is shown in Figure 10, shows that combining the WOFOST model with RS data assimilation provides better LAI simulations than the VI-based empirical method (Figure 5C) and the RS assimilation method (Figure 8C) alone because the problems of leaf aging and error accumulation can be avoided.

The time span delimitation of different crop growth stages is the main limitations for the application of the hybrid method. The time window used for the three crop growth stages in this study influences the application times of the VI-based empirical method, assimilation and crop model without data assimilation. For instance, the beginning of the late-growth stage is the period when reflectance saturation and leaf senescence both appeared; hence, the WOFOST model was used to replace the assimilation method during that period. However, we calculated the start date from the field campaign dates, which may be inaccurate because the goal of the field campaign did not involve measuring reflectance saturation and leaf senescence. More field campaigns should be conducted to acquire the best application time for the three methods.

## 4. Conclusions

In this study, we applied three methods to conduct time-series LAI estimations for spring maize. Using field-measured LAI over an experimental field, the performances of these methods were investigated. Results show that the VI-based method can be used to provide LAI estimation with high accuracy before the appearance of reflectance saturation and leaf senescence. With the application of the UAV data, the accuracy of the assimilation method was improved, and this method was an ideal choice for the mid-stage LAI simulation. The hybrid method is designed to address reflectance saturation and leaf senescence issues of the VI-based method and error accumulation of the WOFOST model in the late stage, and its LAI estimation accuracy of at the late stage is higher than the other two methods. In general, the accuracy analysis results show that the combined methods, which applied the VI-based method for the early stage, the assimilation method for the mid stage, and the hybrid method for the late stage, can provide highly accurate continuous LAI estimations for the entire growth season. Meanwhile, it’s also worth noting that the experiment was only conducted for one year due to the high cost and logistic requirements. The field data gathered in the experiment are insufficient to well evaluated the robustness of the applied methods. A multi-year experiments with additional crop types will be the focus of future studies to further analyze the performance of the combined methods for time-series LAI generation.

## Figures and Tables

**Table 1:** The R values between vegetation indices and field LAI.

Date (Month-Day)	Number of Samples	NDVI	RVI	OSAVI	EVI2	MTVI2
6-30	36	0.78 **	0.79 **	0.75 **	0.67 **	0.73 **
7-29	28	0.63 **	0.64 **	0.55 **	0.46 *	0.39 *
8-25	29	0.30	0.30	0.42 *	0.44 *	0.33

** indicates highly significant correlation, * indicates significant correlation.

**Table 2:** CV values of the vegetation indices for the experimental field (%).

Time (Month-Day)	Field LAI	NDVI	RVI	OSVI	EVI2	MTVI2
6-30	31.51	10.92	30.54	13.44	20.37	20.82
7-29	17.96	1.49	9.35	2.83	6.82	4.12
8-25	12.71	1.51	4.64	2.50	5.38	4.43

**Table 3:** Core parameter calibration results of the WOFOST model.

Parameters	Description	Original Values	Calibrated Values	Unit	Calibration Method
TSUM1	Temperature sum from emergence to anthesis	695	890	°C × d	Field campaign
TSUM2	Temperature sum from anthesis to maturity	800	710	°C × d	Field campaign
CVL	Conversion efficiency of assimilates into leaf	0.68	0.64	kg/kg	Field campaign
CVO	Conversion efficiency of assimilates into storage organ	0.67	0.81	kg/kg	Field campaign
CVR	Conversion efficiency of assimilates into root	0.69	0.70	kg/kg	Field campaign
CVS	Conversion efficiency of assimilates into stem	0.66	0.66	kg/kg	Field campaign
FRTB	Fraction of total dry matter to root	0–0.37	0–0.40	kg/kg	Field campaign
FOTB	Fraction of above ground dry matter to storage organs (DVS = 0.1–1.7)	0–1.00	0–0.74	kg/kg	Field campaign
FLTB	Fraction of above ground dry matter to leaves (DVS = 0.1–1.7)	0–0.62	0.20–0.75	kg/kg	Field campaign
FSTB	Fraction of above ground dry matter to stem (DVS = 0.1–1.7)	0–0.85	0.06–0.57	kg/kg	Field campaign
NBASE	Basic soil nitrogen content	100	40–410	mg/kg	SAN estimation method
PBASE	Basic phosphorus content	100	10–80	mg/kg	SAN estimation method
KBASE	Basic potassium content	100	20–340	mg/kg	Field campaign
SMFCF	Soil moisture content at field capacity	0.11	0.46	cm^3^/cm^3^	FSEOPT software
SMW	Soil moisture content at wilting point	0.04	0.20	cm^3^/cm^3^	FSEOPT software
SM0	Soil moisture content of saturated soil	0.39	0.570	cm^3^/cm^3^	FSEOPT software
RDMCR	Maximum root depth allowed by soil	10	2.4	m	FSEOPT software
SPAN	Life span of leaves growing at 35 °C	33	28	day	FSEOPT software

**Table 4:** Performance of calibrated and un-calibrated WOFOST model.

Variable	Method	Values	Error
Emergence time	Observed	1 June	-
	Original model	23 May	−8 days
	Calibrated model	28 May	−4 days
Anthesis time	Observed results	25 July	-
	Original model	15 July	−10 days
	Calibrated model	29 July	4 days
Maturity time	Observed results	27 September	-
	Original model	22 September	−5 days
	Calibrated model	30 September	3 days
Yield (kg/ha)	Observed results	9179	-
	Original model	9607	−428
	Calibrated model	9104	75
